# Potassium-Enriched Salt Substitutes: A Review of Recommendations in Clinical Management Guidelines

**DOI:** 10.1161/HYPERTENSIONAHA.123.21343

**Published:** 2024-01-29

**Authors:** Xiaoyue Xu, Ling Zeng, Vivekanand Jha, Laura K. Cobb, Kenji Shibuya, Lawrence J. Appel, Bruce Neal, Aletta E. Schutte

**Affiliations:** School of Population Health (X.X., L.Z., A.E.S.), University of New South Wales Sydney, Kensington, Australia.; The George Institute for Global Health (X.X., B.N., A.E.S.), University of New South Wales Sydney, Kensington, Australia.; The George Institute for Global Health, University of New South Wales, New Delhi, India (V.J.).; School of Public Health, Imperial College London, United Kingdom (V.J., B.N.).; Prasanna School of Public Health, Manipal Academy of Higher Education, India (V.J.).; Resolve to Save Lives, New York, NY (L.K.C.).; Tokyo Foundation for Policy Research, Japan (K.S.).; Department of Epidemiology, Bloomberg School of Public Health and Welch Center for Prevention, Epidemiology, and Clinical Research, Johns Hopkins University, Baltimore, MD (L.J.A.).; Hypertension in Africa Research Team, Medical Research Council Unit for Hypertension and Cardiovascular Disease, North-West University, Potchefstroom, South Africa (A.E.S.).; Department of Paediatrics, Medical Research Council/Wits Developmental Pathways for Health Research Unit, Faculty of Health Sciences, University of the Witwatersrand, Johannesburg, South Africa (A.E.S).

**Keywords:** blood pressure, cardiovascular diseases, guidelines, hyperkalemia, hypertension, salts

## Abstract

Excess dietary sodium intake and insufficient dietary potassium intake are both well-established risk factors for hypertension. Despite some successful initiatives, efforts to control hypertension by improving dietary intake have largely failed because the changes required are mostly too hard to implement. Consistent recent data from randomized controlled trials show that potassium-enriched, sodium-reduced salt substitutes are an effective option for improving consumption levels and reducing blood pressure and the rates of cardiovascular events and deaths. Yet, salt substitutes are inconsistently recommended and rarely used. We sought to define the extent to which evidence about the likely benefits and harms of potassium-enriched salt substitutes has been incorporated into clinical management by systematically searching guidelines for the management of hypertension or chronic kidney disease. We found incomplete and inconsistent recommendations about the use of potassium-enriched salt substitutes in the 32 hypertension and 14 kidney guidelines that we reviewed. Discussion among the authors identified the possibility of updating clinical guidelines to provide consistent advice about the use of potassium-enriched salt for hypertension control. Draft wording was chosen to commence debate and progress consensus building: strong recommendation for patients with hypertension—potassium-enriched salt with a composition of 75% sodium chloride and 25% potassium chloride should be recommended to all patients with hypertension, unless they have advanced kidney disease, are using a potassium supplement, are using a potassium-sparing diuretic, or have another contraindication. We strongly encourage clinical guideline bodies to review their recommendations about the use of potassium-enriched salt substitutes at the earliest opportunity.

Higher levels of dietary sodium intake and lower levels of dietary potassium intake are both associated with raised blood pressure (BP) and increased risks of cardiovascular disease and premature death.^1–5^ There is a strong evidence base indicating that both reducing sodium intake and increasing potassium intake will reduce these risks through their BP-lowering effects.^6,7^ As a consequence, multiple authoritative bodies acknowledge these risks^8,9^ and have made recommendations related to sodium and potassium intake. The World Health Organization (WHO), for example, has guidelines that make strong population-wide recommendations to reduce sodium intake and increase potassium intake.^8,9^ For sodium, the WHO has also explicitly proposed that all member states reduce mean population intake by 30% by 2025, with a target maximum intake of 2.0-g/day sodium (5.0-g/day salt),^10^ in support of global efforts to reduce the noncommunicable disease burden by one quarter.

Mean global sodium intake was recently estimated to be 4.3 g/day,^8^ which equates to about 10.8 g/day of table salt. The most recent corresponding estimated value for mean global potassium intake is about 2.3 g/day.^11^ These current intake values are substantially different from norms during hominid evolution when the estimated intake of sodium was about 0.5 g/day and the estimated potassium intake was about 10 g/day. Current sodium consumption varies substantially between populations with the estimated intake highest in the WHO Western Pacific region (6.2-g/day sodium and 15.6-g/day salt) driven by high consumption levels in China and lowest in Africa (2.7-g/day sodium and 6.7-g/day salt; Figure).

**Figure.**
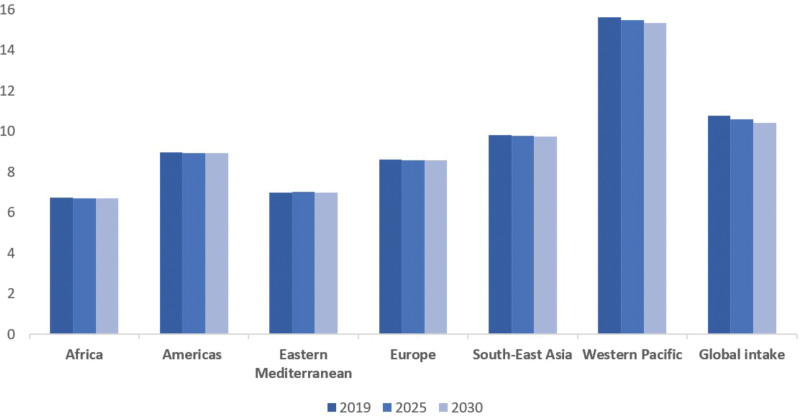
**Estimated mean population dietary salt intake (g/d) by the World Health Organization (WHO) regions in 2019 and projected for 2025 and 2030.** Data from WHO global report on sodium reduction.^8^

Sodium reduction has been identified as a potentially highly cost-effective means for preventing hypertension and cardiovascular diseases around the world.^12–14^ It is listed as a best-buy intervention by the WHO, which has developed the SHAKE (Surveillance, Harness industry, Adopt standards for labelling and marketing, Knowledge, Environment) package to support national sodium reduction efforts.^15^ Multiple countries have commenced implementation of sodium reduction campaigns that include various strategies, for example, consumer education, food voluntary and mandatory reformulation, taxation, and food labeling.^16^ Average global sodium consumption has not, however, fallen over the past decade, and the projected decline over coming years is small—global sodium intake is expected to be 4.23 (10.6-g/day salt) g/day in 2025 and 4.16 (10.4-g/day salt) g/day in 2030 (Figure).^8^

On the contrary, average potassium intake falls significantly below the WHO-recommended intake of 3.5 g/day, as indicated by various studies—≈1.0 g/day in India,^17^ 1.4 g/day in China,^18^ and 1.8 g/day in West Africa.^19^ The reasons for low potassium intake vary but are mainly due to limited agricultural capacity for cultivating crops with high potassium content^20^ and, in particular, limited consumption of potassium-rich fruits and vegetables.^21^ The direct emphasis on increasing dietary potassium intake often lacks guideline recommendations, despite a focus on promoting the consumption of fresh fruits and vegetables (although many are not rich in potassium).^21^ These efforts have largely proven to be ineffective, with many people finding difficulty in adhering to recommendations, in both higher and lower resource settings.^22^ Due to the importance of both sodium and potassium intake, the WHO recommends an intake of <2000 mg of sodium and >3510 mg of potassium daily, resulting in a sodium-to-potassium ratio of ≤1.^23^

The mechanistic rationale for increasing dietary potassium in BP control is explained in recent pretranslational studies.^24,25^ Potassium-rich diets reduce sodium reabsorption along the proximal and distal nephron, analogous to a diuretic, to promote sodium delivery to the collecting duct, which drives potassium secretion and excretion. Reciprocally, low-potassium diets provoke sodium reabsorption and retention to minimize delivery to the collecting duct reducing potassium secretion and excretion.

The chief problem with trying to reduce dietary sodium intake or increase dietary potassium intake is that the required changes by individuals, the food industry, and the government are mostly too difficult to achieve. Despite many efforts over many years, it has proved too hard to change consumer behaviors, reset the palates of populations, modify the food system, or persuade governments to implement regulatory instruments in the face of continued resistance from the food industry. Nonetheless, reducing dietary sodium and increasing dietary potassium remain public health priorities with huge potential for disease prevention. Novel approaches are required to address these priorities.^5^

## POTASSIUM-ENRICHED, SODIUM-REDUCED SALT SUBSTITUTES

Potassium-enriched, sodium-reduced salt substitutes, or potassium-enriched salts, are products that can be used as a direct switch for regular salt (sodium chloride) for seasoning, preserving, and manufacturing foods. Potassium-enriched salts are made by replacing a proportion of the sodium chloride in regular salt with potassium chloride. Sometimes, other nonpotassium substitutes, such as magnesium sulfate, may also be added.^26^ The sodium content of potassium-enriched salts ranges from 0% to 100%.^26^ Other terms used to describe the products include low-sodium salt, potassium salt, mineral salt, and sodium-reduced salt.^26^ The first formal clinical identification of potassium-enriched salt as an option for BP control was included in the 1995 WHO and International Society of Hypertension guidelines, recommending that substitution of common salt by mineral salt low in sodium and rich in potassium and magnesium has been found to be effective in reducing BP in older hypertensives.^27^

A strong body of evidence supports making a like-for-like switch from regular salt to potassium-enriched salt in patients with hypertension.^28^ A key benefit of switching from regular salt to potassium-enriched salt is that it lowers BP through the joint effects of reducing sodium intake and supplementing potassium intake. In addition, potassium-enriched salts seem to be a feasible way of achieving change in sodium and potassium intake in a way that other approaches to reducing sodium and increasing potassium do not. For example, in a recent large-scale and long-term trial that tested the effects of switching to potassium-enriched salt, there was 92% adherence to potassium-enriched salt 5 years after trial commencement with sustained effects objectively demonstrated by 5-year urinary sodium, urinary potassium, and BP levels.^28^ The main reasons why adherence was so high in this and other studies of potassium-enriched salt seem to be that the taste is similar to regular salt and the product can be used like regular salt with no requirement for behavior change in cooking or seasoning.^29–31^

Potassium-enriched salt is also of interest because it can be used to replace regular salt in many food-manufacturing processes including in salty sauces and seasoning that are particularly common in Asian countries.^32^ Potassium-enriched salt could also be switched to regular salt in restaurants and other settings where food is consumed outside of the home. These options are important in those countries, particularly higher income and middle- and high-income Asian countries, where the majority of dietary sodium and potassium intake derives from packaged and restaurant foods.^33^

The effects of potassium-enriched salts on clinical outcomes have been defined in a series of randomized trials.^34^ The most recent systematic review and meta-analyses of 21 trials (31 949 participants) have confirmed the beneficial effects of potassium-enriched salt on a range of clinical outcomes. Across the 19 trials that reported BP outcomes, mean systolic BP was reduced by 4.61 mm Hg and mean diastolic BP was reduced by 1.61 mm Hg. In the 5 trials that reported cardiovascular outcomes, potassium-enriched salt reduced major cardiovascular events by 11%, total mortality by 11%, and cardiovascular mortality by 13%.^34^ Importantly, effects were observed across diverse population subgroups and geographies though the majority of the data were accrued in patients under clinical management for hypertension.

We further explored whether any other trials have been performed after this review (search terms in Table S1). Three trials were identified (Table S2), including the DECIDE (Diet, Exercise and Cardiovascular Health) study in China.^35^ All trials reached the consensus that potassium-enriched salt led to a notable decrease in BP, with the reduction ranging from 4.6 to 7.1 mm Hg for systolic BP and 1.1 to 2.3 mm Hg for diastolic BP.^35–37^ The DECIDE study further reported a 40% reduction in cardiovascular events.^35^

Many of these trials were conducted in regions with notably low dietary potassium consumption, such as rural China^28^ and Peru.^38^ The average benefit of salt substitutes may be less pronounced in countries with higher baseline potassium intake, such as the United States, though benefits will vary substantially between individuals in every country.^39^

The first ever released 2023 WHO Global Report on Hypertension has proposed that potassium-enriched salt is an affordable strategy to reduce BP and prevent cardiovascular events.^40^ Additionally, the recent WHO Global Report on Sodium Intake Reduction suggested that countries explore ways to increase the availability and use of potassium-enriched, sodium-reduced salt substitutes for BP control and other health benefits.^8^ At the same time, the US Food and Drug Administration is updating regulations to support the use of salt substitutes as a mechanism to reduce sodium content in processed foods.^41^ As for sodium reduction, several studies project that salt substitutes have the potential to be a cost-effective approach to lowering BP at a population level.^42,43^

## HYPERTENSION MANAGEMENT GUIDELINE RECOMMENDATIONS ON POTASSIUM-ENRICHED SALT SUBSTITUTES

There has been long-standing recognition of the importance of sodium in the causation of high BP, and therefore, hypertension management guidelines have for many iterations included recommendations for dietary sodium reduction. Some, though many fewer, have also made recommendations related to enhancing dietary potassium consumption. Less clear is the extent to which recommendations relating to the use of potassium-enriched salt have been included. Accordingly, we performed a review of global, regional, and national hypertension guidelines by searching the databases of Medline and Web of Science for guidelines published between January 1, 2013, and June 21, 2023 (Table S3). We also screened the reference lists of relevant publications and retrieved a total of 32 hypertension management guidelines from the initial 847 hits returned by the searches. The search process is documented in a PRISMA (Preferred Reporting Items for Systematic Reviews and Meta-Analyses) flowchart (Figure S1).

The 32 guidelines identified included those from 2 global organizations, 5 regional organizations, and 25 country organizations (Table 1). All were searched for recommendations related to dietary sodium reduction, dietary potassium supplementation, and the use of potassium-enriched salts. All included specific reference to sodium reduction, with most recommending to reduce salt intake to a level between 4 and 6 g/day for the primary or secondary prevention of cardiovascular disease (Table 1).^44,46–50,52–55,57–59,62,63,65,66,68–72,74,75^ Many made specific recommendations on dietary potassium intake,^9,45,46,48,51–60,62–67,70^ and 4—the Chinese, European, Taiwanese, and British—made specific mention of potassium-enriched salt (Table 2).^46,52,60,63^ Of those, 2 made a specific recommendation for the use thereof,^46,63^ one simply mentioned the recently completed SSaSS trial (Salt Substitute and Stroke Study),^52^ and one included only a warning that potassium-enriched salt should not be used in those in whom it may be contraindicated.^60^

**Table 1.**
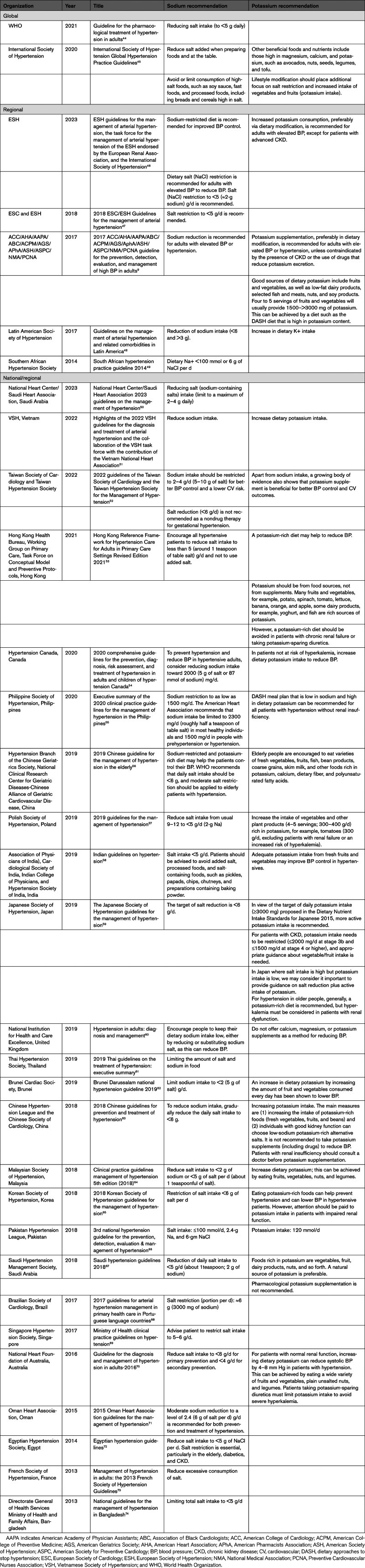
Sodium and Potassium Recommendations in Global, Regional, and National Hypertension Management Guidelines

**Table 2.**
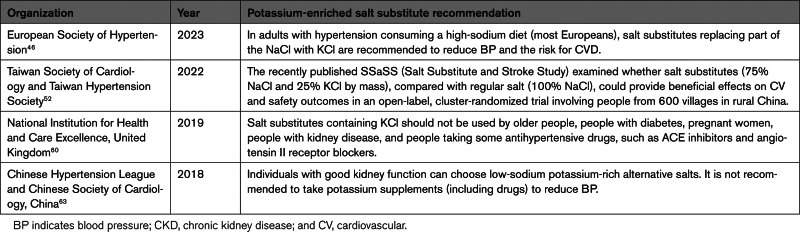
Potassium-Enriched Salt Recommendations in Hypertension Management Guidelines

## POTASSIUM-ENRICHED SALTS AND THE RISK OF HYPERKALEMIA

A frequently raised concern about the use of potassium-enriched salts is the risk of hyperkalemia. This is raised particularly in the context of patients with chronic kidney disease, where there is a longstanding advice to avoid dietary potassium, but, sometimes, more broadly to other population subgroups. The National Institution for Health and Care Excellence in the United Kingdom,^60^ for example, advises that salt substitutes containing potassium chloride should not be used by older people, people with diabetes, pregnant women, people with kidney disease, and people taking some antihypertensive drugs, such as ACE inhibitors and angiotensin II receptor blockers.

The case for avoiding potassium-enriched salt in the presence of advanced kidney disease is widely accepted, but the rationale for broad contraindications like those applied in the United Kingdom is unclear. The most recent review of trials of potassium-enriched salt^34^ identified no effect on hyperkalemia risk. Salt substitute–induced hyperkalemia has been identified in the recently reported DECIDE salt study though sustained elevations were uncommon and there were no adverse effects associated with the observed elevations.^35^ Of note, the DECIDE salt design included people regardless of potential hyperkalemia risk, whereas all other studies have used some form of screening to avoid use in those potentially contraindicated. In SSaSS, the largest trial to collect safety data related to potassium-enriched salts, the highly pragmatic design meant that there was no information about the occurrence of biochemical hyperkalemia, but the rate of serious adverse events attributed to hyperkalemia was not higher with potassium-enriched salt compared with regular salt. Neither was there any increased risk of sudden cardiac death that might be attributed to hyperkalemia-induced arrhythmia.^28^ Because most trials took steps to exclude participants at elevated risk of hyperkalemia, primarily those with advanced kidney disease or using medications that elevate serum potassium, good data about the effects of potassium-enriched salt in these groups are not available.^34,76^ In some, including SSaSS, exclusion was, however, based only on patient reports with no direct measurement of kidney function suggesting that population-wide use may be safe and effective.

## MANAGEMENT GUIDELINES FOR CHRONIC KIDNEY DISEASE AND RECOMMENDATIONS ON POTASSIUM-ENRICHED SALT SUBSTITUTES

Given long-standing clinical advice to restrict dietary potassium consumption among patients with chronic kidney disease, we also investigated chronic kidney disease management guidelines for recommendations related to potassium-enriched salt. In parallel, we also extracted data referencing dietary sodium and dietary potassium. Once again, we searched the databases of Medline and Web of Science for relevant guidelines published between January 1, 2013, and June 21, 2023 (Table S4), and, as before, screened the reference lists of relevant publications for additional data sources. We identified a total of 14 chronic kidney disease management guidelines (Table 3) and documented the search process in a PRISMA flowchart (Figure S2).

**Table 3.**
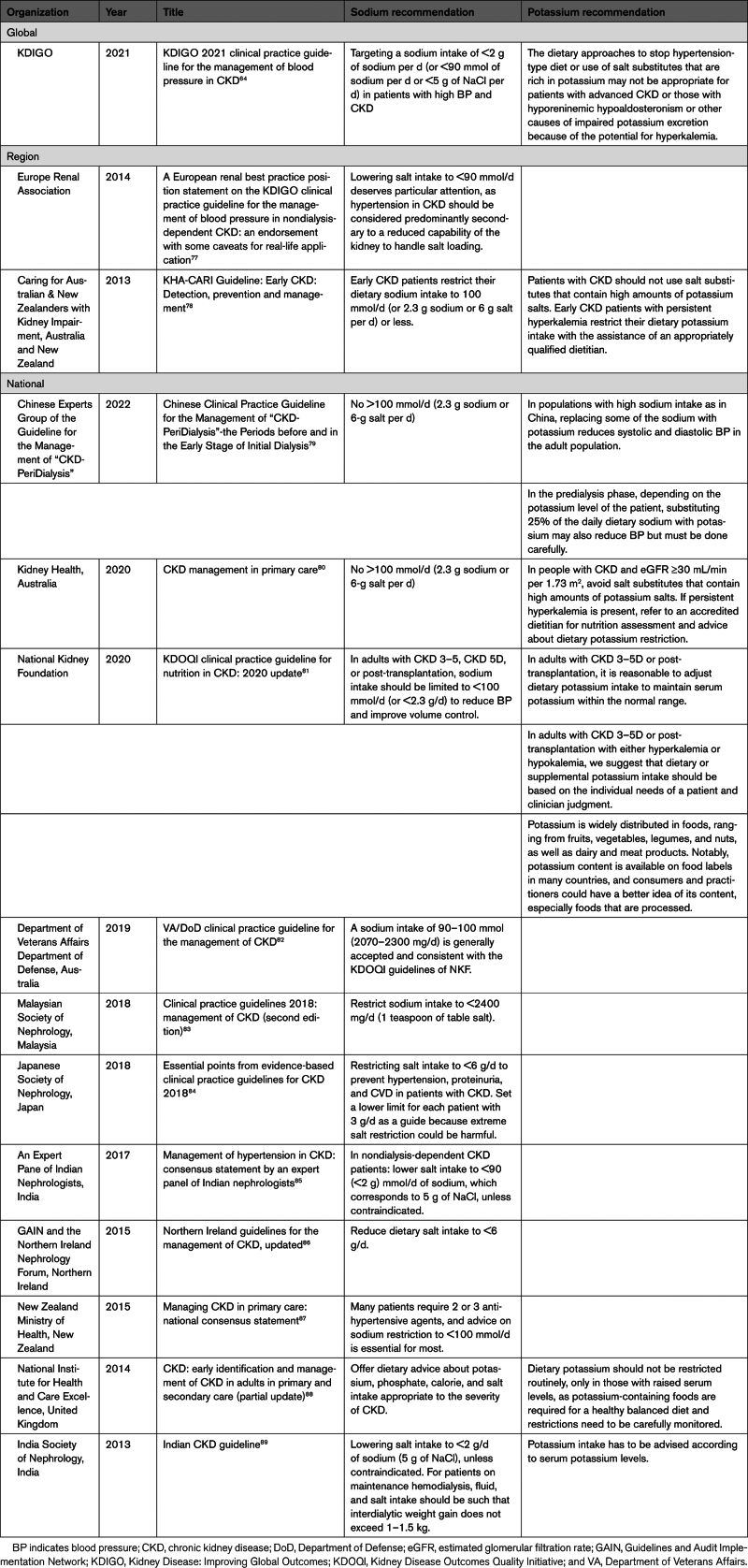
Sodium and Potassium Recommendations in Global, Regional, and National CKD Management Guidelines

Of the 14 guidelines, there was 1 from a global organization, 2 from regional organizations, and 11 from national organizations. All the guidelines stressed sodium restriction^76–89^ (Table 3) though some, such as the Japanese Society of Nephrology, set a lower limit below which sodium intake should not be further reduced because of perceived risks of harm.^84^ However, a recent meta-analysis of studies classifying sodium intake with 24-hour urine collections found a direct linear association between sodium intake and cardiovascular events, demonstrating that sodium restriction is safe.^90^ Seven guidelines provided advice about the value of a potassium-restricted diet to avoid hyperkalemia (Table 3).^76,78–81,88,89^ Only 4 of these management guidelines specifically mentioned potassium-enriched salt (Table 4). Three guidelines^76,78,80^ warned that salt substitutes rich in potassium are not recommended for patients with CKD, while the fourth, the Chinese Clinical Practice Guideline,^79^ advised of potential benefit from careful use of salt substitutes during the predialysis phase. The Kidney Disease Improving Global Outcomes guideline advises exercising caution when using salt substitutes in individuals with advanced CKD of stage 4 and stage 5 but not for individuals at early stages^76^ (Table 4).

**Table 4.**
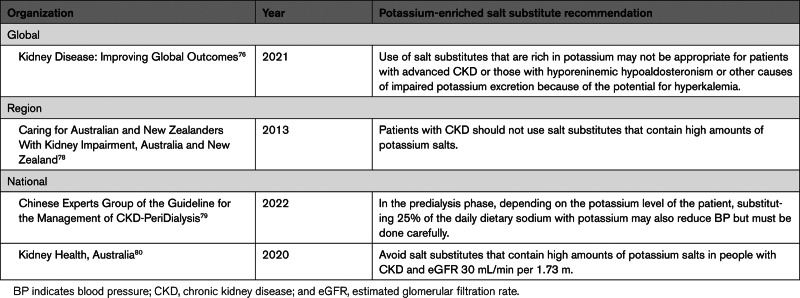
Potassium-Enriched Salt Recommendations in CKD Management Guidelines

## ALIGNING THE EVIDENCE WITH GUIDELINE RECOMMENDATIONS FOR USE OF POTASSIUM-ENRICHED SALT

The evidence base leaves little doubt that switching from regular salt to potassium-enriched salt will lower BP in patients with hypertension by jointly reducing sodium intake and increasing potassium intake.^91^ It is also clear that the BP reduction achieved with potassium-enriched salt will protect against serious complications of hypertension, such as stroke and premature death. Furthermore, the overviews of the trials suggest that a BP-lowering effect will be achieved across diverse subsets of people with hypertension though the magnitude of the fall will vary according to factors such as the starting BP, the baseline levels of sodium and potassium consumption, and how much dietary salt consumption can be switched. Given that potassium-enriched salt is one of the few dietary interventions that patients are likely to be able to adhere to long term, it is logical for all patients with hypertension to be considered for the use of potassium-enriched salt.

At the same time, it is important that patients with hypertension are not harmed by a potassium-enriched salt, so contraindications to use must be part of any recommendation on their use. These should include the presence of advanced kidney disease and the concomitant use of potassium-sparing diuretics or potassium supplements. However, it is almost certainly not appropriate to exclude all older people, all people with diabetes, and all people taking antihypertensive drugs, such as ACE inhibitors and angiotensin II receptor blockers. Unless there is an undiagnosed intercurrent advanced kidney disease that results in misuse of potassium-enriched salt, most will have a low risk of hyperkalemia, which will be outweighed by a high likelihood of benefiting from additional BP reduction. Many older people, people with diabetes, and people taking antihypertensive drugs, such as ACE inhibitors and angiotensin II receptor blockers, have difficulty in controlling hypertension and were observed to achieve benefits, without any evidence of harm, in the SSaSS trial.^28^

Data to directly support the use of potassium-enriched salt outside the clinical hypertension setting are more limited though the totality of the available evidence suggests that the use of potassium-enriched salt would also reduce BP among the general population. The chief concern about the population-wide use of potassium-enriched salt relates to the possibility that individuals with undiagnosed advanced kidney disease might use potassium-enriched salt and develop hyperkalemia as a consequence. While trials that test this question directly have not been done, high-quality modeling studies suggest a large net benefit from population-wide use of potassium-enriched salt, even under worst-case assumptions about harm from hyperkalemia.^43,92^ The benefit-risk balance is defined primarily by the high global prevalence of hypertension (about 32% of adults),^93^ the low prevalence of chronic kidney disease (about 10%^94^ with only 2% at late-stage end-stage kidney disease),^95^ and the potential for patients with chronic kidney disease to benefit from BP lowering, not just be harmed by hyperkalemia. Other data also suggest that concerns about hyperkalemia from dietary potassium consumption may be overestimated.^96,97^ A 2023 study of 367 patients with stage 1 to 4 chronic kidney disease reported no association between dietary potassium consumption and blood potassium levels.^97^ This is a finding directly comparable to that observed in 212 patients with dialysis- and nondialysis-dependent chronic kidney disease reported a few years earlier.^98^

## RECOMMENDED TEXT FOR INCLUSION IN CLINICAL MANAGEMENT GUIDELINES

The adoption of agreed standardized wording to describe recommendations for the use of potassium-enriched salt would provide consumers, clinicians, and governments worldwide reassurance about the best practices. To this end, we have drafted boilerplate text that can form the basis for discussion about updates to clinical management guidelines worldwide. This text will be shared with guideline groups to seek input and achieve widespread clinical use.

## RECOMMENDED STANDARD WORDING FOR GUIDANCE ABOUT THE USE OF POTASSIUM-ENRICHED SALT IN CLINICAL MANAGEMENT GUIDELINES

### Strong Recommendation for Patients With Hypertension

Potassium-enriched salt with a composition of ≈75% sodium chloride and 25% potassium chloride should be recommended to all patients with hypertension, unless they have advanced kidney disease, are using a potassium supplement, are using a potassium-sparing diuretic, or have another contraindication.

### Conditional Recommendation for the General Population

If you have to add salt to foods, potassium-enriched salt with a composition of ≈75% sodium chloride and 25% potassium chloride can be recommended for use by the general population in settings where there is a low likelihood that people with advanced kidney disease (stages 4 and 5) will be undiagnosed by the health system and contraindications to use can be printed on product packaging.

The strong recommendation for use in patients with hypertension is underpinned by the premise that the clinical contact inherent in the management of hypertension will make it possible to control the risk of hyperkalemia.^34,35^ The conditional recommendation for use in the general population depends on there being clear enunciation of the contraindications to use on the package labeling.^99^

It is important to increase the production capacity and align with the growing market demand for salt substitutes. Key stakeholders, such as the American Heart Association and the American Society of Nephrology, and also global organizations are strongly encouraged to engage with manufacturers and provide heart-healthy recommendations for using potassium-enriched salt substitutes as alternatives to traditional salt (including in processed, packaged, and prepared foods). By replacing traditional salt with potassium-enriched salt substitutes in the household, the cumulative protective effects are likely applicable across the entire life course and may lead to enhanced BP control from childhood and the prevention of cardiovascular disease into adulthood.

## CONCLUSIONS

A strong body of evidence supports the replacement of regular salt with potassium-enriched salt in patients with hypertension. There is also a case for the general population making the switch to potassium-enriched salt where risks of misuse can be managed. Current clinical guidelines offer incomplete and inconsistent recommendations about the use of potassium-enriched salt substitutes, as well as reducing dietary sodium intake and increasing dietary potassium intake. We urge all relevant clinical guideline bodies to debate the value of potassium-enriched salt as a routine adjunct to drug therapy and update their recommendations accordingly. Evidence suggests that there are likely to be substantial benefits from the much wider use of potassium-enriched salt with a composition of 75% sodium chloride and 25% potassium chloride, by patients with hypertension. As part of their updates, clinical guideline bodies should provide consistent recommendations about the use of potassium-enriched salt substitutes and actively promote these recommendations to their constituents.

## ARTICLE INFORMATION

### Sources of Funding

X. Xu was supported by the Heart Foundation Postdoctoral Fellowship funded by the Heart Foundation of Australia (award No. 102597) and the Scientia Program at the University of New South Wales, Australia. A.E. Schutte was supported by an National Health and Medical Research Council Leadership Investigator Grant (Application ID: 2017504). B. Neal was supported by an NHMRC Investigator Grant (Application ID: 1197709). L. Appel was supported by Resolve to Save Lives that is funded by Bloomberg Philanthropies, the Bill and Melinda Gates Foundation, and Gates Philanthropy Partners, which is funded with support from the Chan Zuckerberg Foundation.

### Disclosures

L.J. Appel receives payments from Wolters Kluwer for chapters in UpToDate on the relation of blood pressure with weight, exercise, smoking, and sodium intake. The other authors report no conflicts.

## Supplementary Material


