# Supplementary material for: Potassium-Enriched Salt Substitutes: A Review of Recommendations in Clinical Management Guidelines
Source: Hypertension. 2024 Jan 29;81(3):400–14. doi: 10.1161/HYPERTENSIONAHA.123.21343 (PMC10863666; doi:10.1161/HYPERTENSIONAHA.123.21343)
Supplement: Supplementary file 1 [file hyp-81-400-s001.docx]

**Potassium-enriched salt substitutes: a review of recommendations in clinical management guidelines**

Xiaoyue Xu^1,2^, Ling Zeng^1^, Vivekanand Jha^3,4,5^, Laura Cobb^6^, Kenji Shibuya^7^, Lawrence Appel^8,9^, Bruce Neal^2,4^, Aletta E. Schutte^1,2,10,11^

1. School of Population Heath, UNSW Sydney, Australia

2. The George Institute for Global Health, UNSW Sydney, Australia

3. The George Institute for Global Health, UNSW, New Delhi, India

4. School of Public Health, Imperial College London, United Kingdom

5. Prasanna School of Public Health, Manipal Academy of Higher Education, Manipal, India

6. Resolve to Save Lives, Vital Strategies, New York, USA

7. Tokyo Foundation for Policy Research, Tokyo, Japan

8. Department of Epidemiology, Bloomberg School of Public Health, Johns Hopkins University, Baltimore, Maryland, USA

9. Welch Center for Prevention, Epidemiology, and Clinical Research, Johns Hopkins University, Baltimore, Maryland, USA

10. Hypertension in Africa Research Team, MRC Unit for Hypertension and Cardiovascular Disease, North-West University, South Africa

11. SAMRC/Wits Developmental Pathways for Health Research Unit, Department of Paediatrics, Faculty of Health Sciences, University of the Witwatersrand, South Africa

**Short title:** Adopting potassium-enriched salt substitutes in clinical guidelines

**Corresponding author**

Professor Aletta E Schutte

School of Population Health, University of New South Wales, Kensington Campus, High Street, Sydney, NSW 2052, Australia

E-mail: [a.schutte@unsw.edu.au](mailto:a.schutte@unsw.edu.au) ; Tel: +61 450 315 918

**Table S1. Search terms of understanding the effect of salt substitute on clinical outcomes**

| **Database** | **Search terms** | **Filters** | **Search outcome** |
| --- | --- | --- | --- |
| Medline | "salt substitute*" or "low sodium adj5 salt" or "sodium free adj5 salt" or "salt replac*" or "mineral adj5 salt" or "smart salt" or "sodium adj3 reduced adj5 salt" or "health* salt" or "potassium* adj5 salt" or "lite salt" Or *sodium chloride/ Or potassium chloride/ | ab,ti  Ct (Clinical trial) | 75 |
| Embase | "salt substitute*" or "low sodium adj5 salt" or "sodium free adj5 salt" or "salt replac*" or "mineral adj5 salt" or "smart salt" or "sodium adj3 reduced adj5 salt" or "health* salt" or "potassium* adj5 salt" or "lite salt" Or *sodium chloride/ Or potassium chloride/ | ab,ti  Ct (Clinical trial) | 189 |
| Cochrane | "salt substitute*" or "low sodium NEAR/5 salt" or "sodium free NEAR/5 salt" or "salt replac*" or "mineral NEAR/5 salt" or "smart salt" or "sodium NEAR/3 reduced NEAR/5 salt" or "health* salt" or "potassium* NEAR/5 salt" or "lite salt" Or sodium chloride Or potassium chloride | Trial | 91 |

**Table S2. Recent clinical trials on the effects of salt substitutes on blood pressure and clinical outcomes, since publication of a 2021 meta-analysis^31^**

| **Study settings** | **Sample** | **Mean follow-up** | **Comparisons** | **Outcome measures** | **Effects** |
| --- | --- | --- | --- | --- | --- |
| China ^32^ | 1,612 participants aged 55 years or older | 2 years | Salt substitute (25% potassium) vs regular salt | BP, cardiovascular events and total mortality. | Lowered SBP by 7.1 mmHg, DBP by 1.9 mmHg; reduced 40% of cardiovascular events but no effect on total mortality. |
| India ^33^ | 502 participants with hypertension (aged 61.6 ± 12 years) | 3 months | Salt substitute (70% sodium chloride/30% potassium chloride blend) vs regular salt (100% sodium chloride) | BP | Reduced average SBP by 4.6 mmHg and average DBP by 1.1 mmHg. |
| China ^34^ | 322 middle-aged and elderly hypertensive patients | 12 months | Low-sodium salt vs normal salt | Office and home BP | Reduced office SBP and DBP by 6.6 and 1.8 mmHg; Reduced home SBP by 4.6 mmHg and DBP by 2.3 mmHg. |

**Table S3. Keywords searching for Hypertension Management Guidelines**

| Database | Search terms | Filter | Result |
| --- | --- | --- | --- |
| Medline | (hypertension or "blood pressure").ti.  AND  (management or practice or treatment or prevention).ti.  AND  (guideline* or statement* or societ*).ti. | 2013 to current (21 June 2023) | 635 |
| Web of Science | (hypertension or "blood pressure")  AND  (management or practice or treatment or prevention)  AND  (guideline* or statement* or societ*) | Search in title  2013 to 2023 | 845 |

**Figure S1. PRISMA Chart of searching Hypertension Management Guidelines**

**
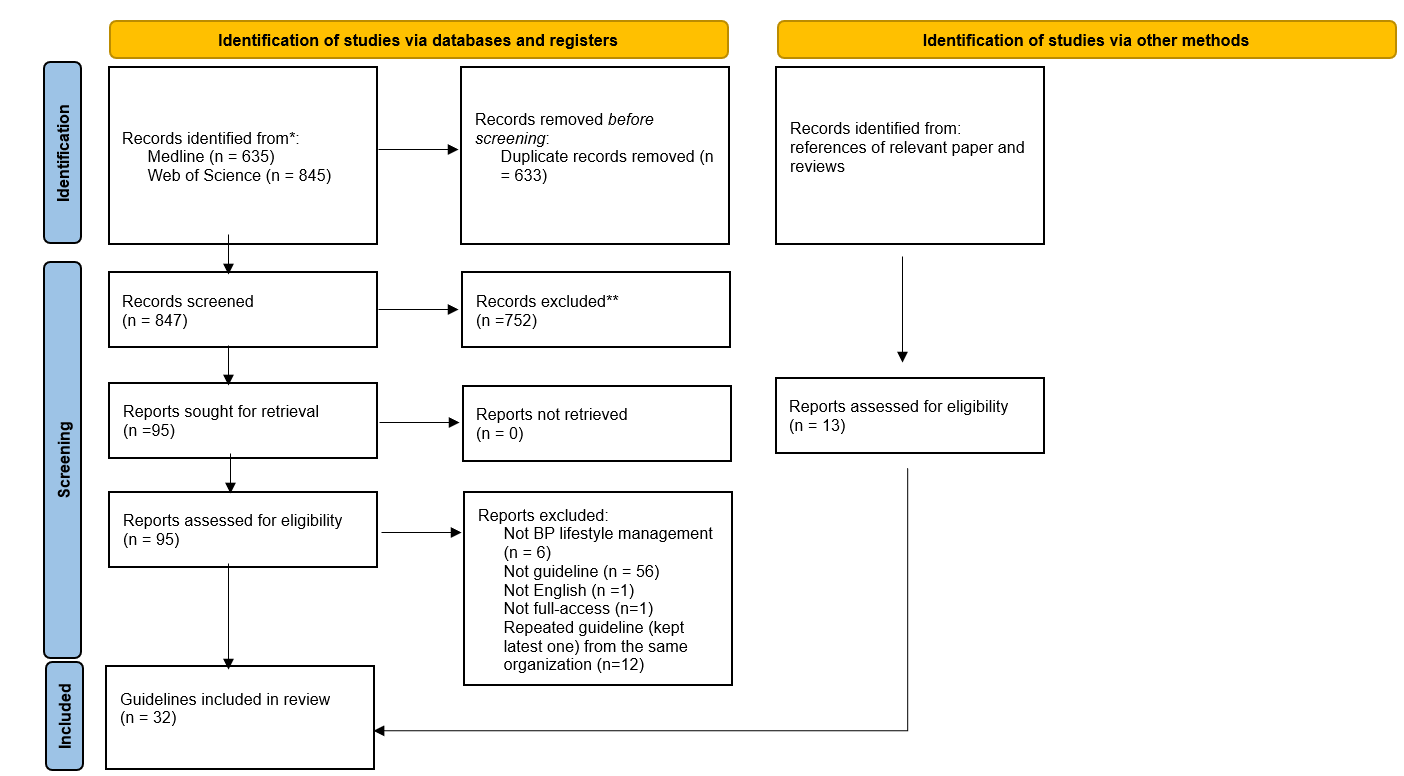
**

**Table S4. Key words searching for chronic kidney disease management guideline**

| Database | Search terms | Filter | Result |
| --- | --- | --- | --- |
| Medline | (“chronic kidney disease” or “chronic renal disease”)  AND  (management or practice or treatment)  AND  (guideline* or statement* or societ*or association*) | 2013 to current (13 June 2023) | 125 |
| Web of Science | (“chronic kidney disease” or “chronic renal disease”)  AND  (management or practice or treatment)  AND  (guideline* or statement* or societ*or association*) | Search in title  2013 to 2023 | 181 |

**Figure S2. PRISMA chart of searching Chronic Kidney Disease Management Guidelines**

**
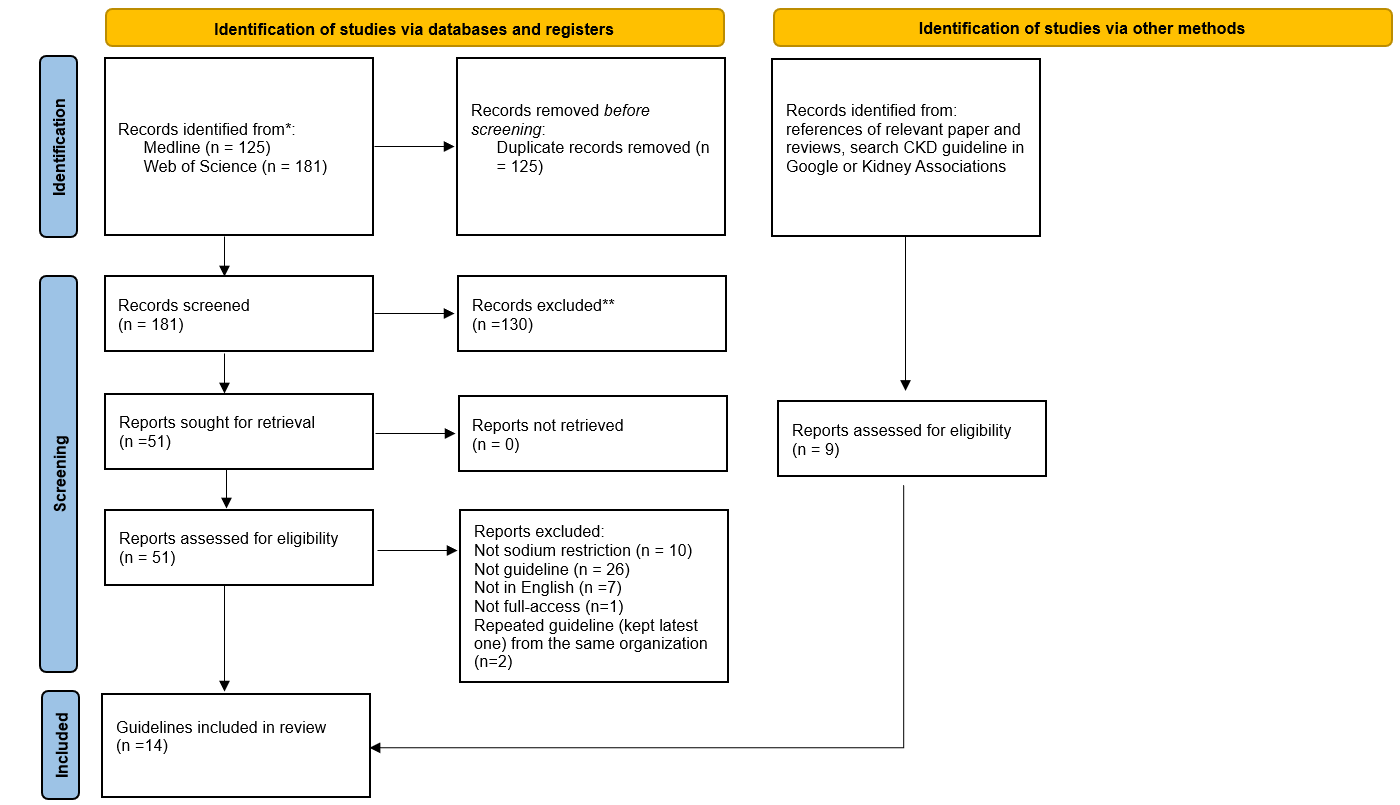
**
